# Left bundle branch area pacing in a case of Steinert’s disease

**DOI:** 10.1016/j.hrcr.2025.01.001

**Published:** 2025-01-07

**Authors:** José M. Sánchez-Moreno, Laura Valverde Soria, Rosa Macías Ruiz, Luis Tercedor, Juan Jiménez-Jáimez, Manuel Molina-Lerma

**Affiliations:** Arrythmia Unit, Cardiology Department, Hospital Universitario Virgen de las Nieves, Granada, Spain

**Keywords:** Steinert’s disease, Myotonic dystrophy type 1, Left bundle branch block, Conduction system pacing, Physiological pacing, LBBAP

**Key Teaching Points**

• Myotonic dystrophy type 1 often presents with cardiomyopathy and conduction disease, especially impairment in the His-Purkinje system.
• In patients with neuromuscular diseases, such as myotonic dystrophy type 1 and any second- or third-degree atrioventricular block, with or without symptoms, permanent pacing is indicated.
• Left bundle branch area pacing could be a feasible and more physiological pacing alternative in patients with Steinert’s disease.

## Introduction

Myotonic dystrophy type 1 (DM1), or Steinert’s disease, is a neuromuscular myotonic disorder involving the endocrine system, central nervous system, eyes, gastrointestinal system, and heart, and is associated with the presence of abnormal expansion of a cytosine-thymine-guanine trinucleotide repeat on chromosome 19q13.3.^1^ DM1 often presents with cardiomyopathy and conduction disease, especially impairment in the His-Purkinje system.^2^

Permanent pacing is indicated in patients with neuromuscular diseases, such as DM1, and any second- or third-degree atrioventricular block, with or without symptoms. In asymptomatic cases with HV interval >70 ms, a pacemaker implantation may be recommended in spite of the lack of evidence.^3^ Conduction system pacing (CSP), including His bundle pacing (HBP) and left bundle branch area pacing (LBBAP), which encompasses left bundle branch pacing, left fascicular pacing, and left ventricular septal pacing,^4^ has been accepted as a feasible and safe pacing strategy for those requiring antibradycardia treatment,^5^ even in the case of infra-Hisian block, demonstrating significantly reduced adverse clinical outcomes.^6^

There is increasing interest in physiological pacing techniques that directly activate the specialized conduction system. With LBBAP, the lead is implanted slightly distal to the His bundle and is screwed deep in the interventricular septum, ideally to capture the left bundle branch.

## Case report

**Figure 1:**
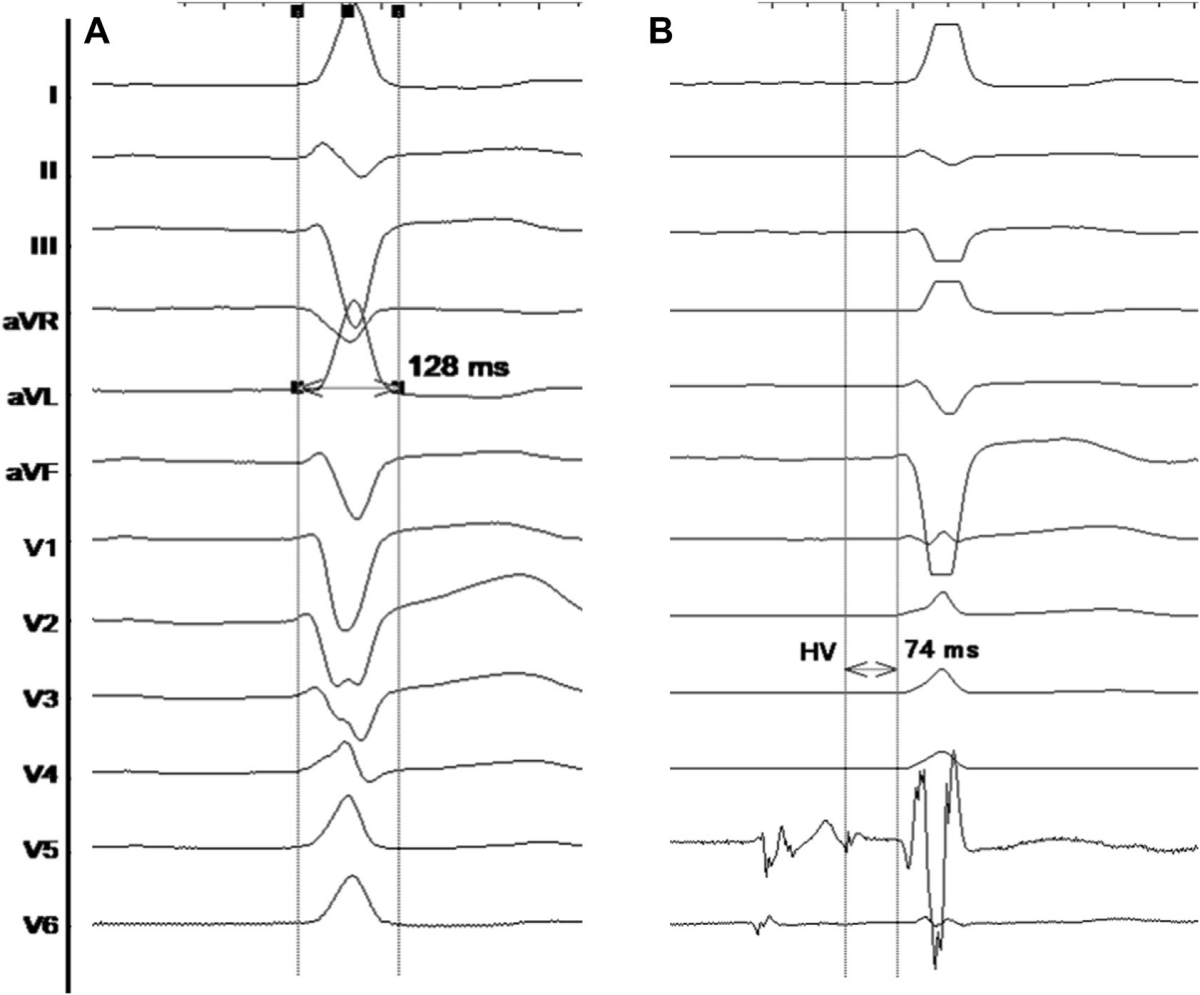
**A:** Basal QRS complex with left bundle branch block morphology (duration 128 ms). **B:** HV interval measured (74 ms) in the electrophysiological study.

A 50-year-old man with DM1, first-degree atrioventricular block and left bundle branch block, which did not fulfill the Strauss criteria,^7^ with QRS duration of 128 ms (Figure 1A) was referred for electrophysiological study. He had no history of syncope, palpitations, or documented arrhythmias. Transthoracic echocardiography showed normal biventricular ejection fraction without any pathologic findings. A pathologic HV interval of 74 ms was observed (Figure 1B). Therefore, pacemaker implantation was indicated.

**Figure 2:**
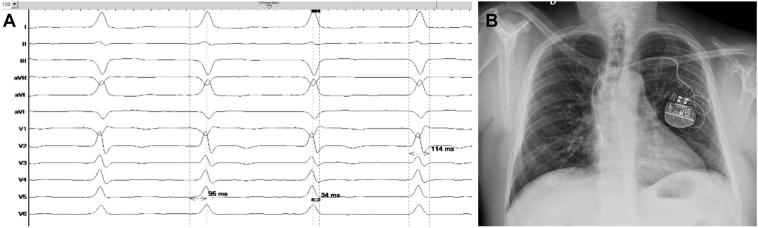
**A:** Paced QRS complex after nonselective left bundle branch pacing with a left ventricular activation time of 96 ms and an interpeak interval of 34 ms. **B:** Anteroposterior radiograph chest view demonstrating the lead position in the basal septum.

**Figure 3:**
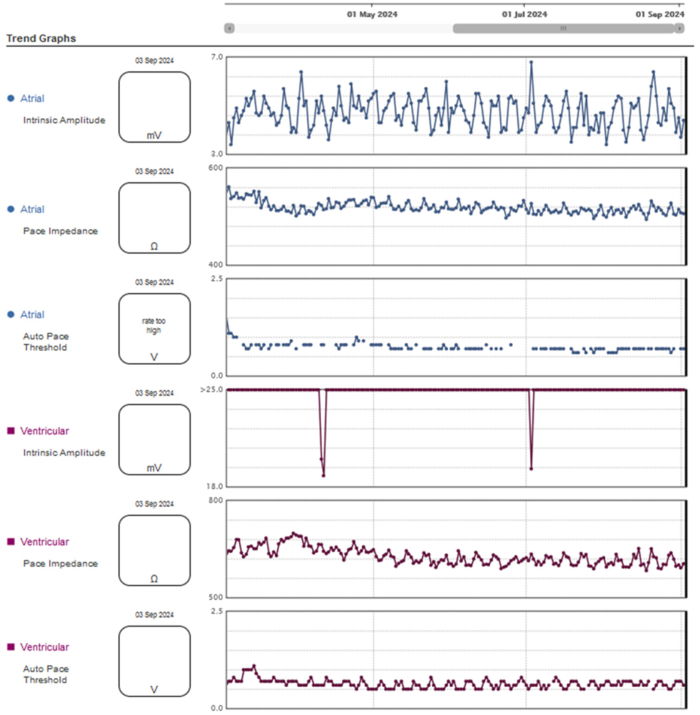
Remote monitoring after 6-month follow-up.

Following local anesthesia, double left axillary vein puncture was performed. A SelectSecure™ MRI SureScan™ Model 3830 pacing lead (Medtronic, Minneapolis, MN) was inserted with the support of a preshaped catheter (model C315HIS, Medtronic) in the left ventricle septum, achieving nonselective left bundle branch pacing (left ventricular activation time of 96 ms and interpeak interval of 34 ms). Pacing threshold, impedance, and mean R-wave amplitude were 0.8 V at 0.4 ms, 934 Ω, and >25 mV, respectively. Afterwards, the atrial lead was placed into the right atrial appendage. Electrocardiography showed a paced QRS duration of 114 ms with unipolar pacing (Figure 2A). DDD mode was programmed. No periprocedural complications were observed and chest radiograph showed adequate lead position with no signs of acute pneumothorax (Figure 2B). At follow-up visits, the patient remained asymptomatic, ejection fraction was preserved, and pacemaker parameters remained stable (Figure 3).

## Discussion

It is widely accepted that conduction disease observed in patients with DM1 is commonly due to impairment in the His-Purkinje system. However, its pathogenesis is not clear. Although histologic studies of the heart in DM1 are scarce, common findings are interstitial fibrosis and fatty infiltration of the myocardium.^8^ A possible concern regarding CSP feasibility in these patients might be the risk of inefficient pacing because of severe conduction disease distal to the stimulation point. Nonetheless, previous published studies suggest otherwise since the target in LBBAP is more distal and broader than that in HBP.^9^ It has been demonstrated that HBP and LBBAP can effectively improve cardiac function by successfully correcting left bundle branch block in patients with heart failure.^10^ Postulated mechanism for these findings are source-sink mismatch, where an increase in source power can facilitate depolarization of distal tissue, and virtual electrode polarization, where an electrical stimulus generates regions of depolarization or hyperpolarization around the electrode tip, which in turn creates potential pathways for propagation through excitation of previously refractory tissue.^11^

Our experience with LBBAP in a patient with DM1 does not differ when using this approach in patients with left bundle branch block related to other conditions. In a recent systematic review and meta-analysis,^12^ patients with indications for cardiac resynchronization therapy followed up for 6–27 months presented better clinical and functional outcomes with CSP than conventional biventricular pacing. Hence, LBBAP could also be a feasible and more physiological pacing alternative for patients with DM1. However, long-term follow-up is needed to ensure its safety and durability because progressive degeneration of conduction system disease is described in DM1. To our knowledge, this is the first published case of LBBAP in a patient with DM1, and more evidence is needed to clarify the best pacing strategy for this subgroup of patients.

## Disclosures

The authors have no conflicts of interest to disclose.
